# Gp96 Peptide Antagonist gp96-II Confers Therapeutic Effects in Murine Intestinal Inflammation

**DOI:** 10.3389/fimmu.2017.01531

**Published:** 2017-12-11

**Authors:** Claudia A. Nold-Petry, Marcel F. Nold, Ofer Levy, Yossef Kliger, Anat Oren, Itamar Borukhov, Christoph Becker, Stefan Wirtz, Manjeet K. Sandhu, Markus Neurath, Charles A. Dinarello

**Affiliations:** ^1^Ritchie Centre, Hudson Institute of Medical Research, Clayton, VIC, Australia; ^2^Department of Paediatrics, Monash University, Melbourne, VIC, Australia; ^3^Compugen Ltd., Holon, Israel; ^4^Medical Clinic 1, Friedrich Alexander University Erlangen-Nuremberg, Erlangen, Germany; ^5^Department of Gastroenterology, Monash Health, Clayton, VIC, Australia; ^6^Department of Medicine, University of Colorado Denver, Aurora, CO, United States

**Keywords:** Gp96, cytokines and inflammation, biologics, therapeutics, immunemodulatory, anti-inflammatory agent, intestinal inflammation, inflammatory bowel disease

## Abstract

**Background:**

The expression of heat shock protein gp96 is strongly correlated with the degree of tissue inflammation in ulcerative colitis and Crohn’s disease, thereby leading us to the hypothesis that inhibition of expression *via* gp96-II peptide prevents intestinal inflammation.

**Methods:**

We employed daily injections of gp96-II peptide in two murine models of intestinal inflammation, the first resulting from five daily injections of IL-12/IL-18, the second *via* a single intrarectal application of TNBS (2,4,6-trinitrobenzenesulfonic acid). We also assessed the effectiveness of gp96-II peptide in murine and human primary cell culture.

**Results:**

In the IL-12/IL-18 model, all gp96-II peptide-treated animals survived until day 5, whereas 80% of placebo-injected animals died. gp96-II peptide reduced IL-12/IL-18-induced plasma IFNγ by 89%, IL-1β by 63%, IL-6 by 43% and tumor necrosis factor (TNF) by 70% compared to controls. The clinical assessment Disease Activity Index of intestinal inflammation severity was found to be significantly lower in the gp96-II-treated animals when compared to vehicle-injected mice. gp96-II peptide treatment in the TNBS model limited weight loss to 5% on day 7 compared with prednisolone treatment, whereas placebo-treated animals suffered a 20% weight loss. Histological disease severity was reduced equally by prednisolone (by 40%) and gp96-II peptide (35%). Mice treated with either gp96-II peptide or prednisolone exhibited improved endoscopic scores compared with vehicle-treated control mice: vascularity, fibrin, granularity, and translucency scores were reduced by up to 49% by prednisolone and by up to 30% by gp96-II peptide. *In vitro*, gp96-II peptide reduced TLR2-, TLR4- and IL-12/IL-18-induced cytokine expression in murine splenocytes, with declines in constitutive IL-6 (54%), lipopolysaccharide-induced TNF (48%), IL-6 (81%) and in *Staphylococcus epidermidis*-induced TNF (67%) and IL-6 (81%), as well as IL-12/IL-18-induced IFNγ (75%). gp96-II peptide reduced IL–1β, IL-6, TNF and GM-CSF in human peripheral blood mononuclear cells to a similar degree without affecting cell viability, whereas RANTES, IL-25 and MIF were twofold to threefold increased.

**Conclusion:**

gp96-II peptide protects against murine intestinal inflammation by regulating inflammation *in vivo* and *in vitro*, pointing to its promise as a novel treatment for inflammatory bowel disease.

## Introduction

Inflammatory bowel disease (IBD), which includes Crohn’s disease (CD) and ulcerative colitis (UC), is characterized by intense and chronic inflammation of the intestinal tract that damages the epithelium and allows penetration of bacteria across the gut epithelial barrier. Subsequently, T cells infiltrate (1) and release alarmins or damage-associated molecular patterns (DAMPs) such as gp96.

Gp96 is a multifunctional eukaryotic endoplasmic reticulum (ER) heat shock protein (HSP) that is expressed constitutively in virtually all cell types and its synthesis is increased in conditions causing ER stress (2). gp96 plays a chaperone role for most of the toll-like receptors (TLRs) (3, 4) that are essential for the innate immune response recognizing microbial products. In addition to its role in innate immunity, gp96 activates the adaptive immune pathways. After tissue damage or viral infection, gp96 is released into the extracellular space and exerts immune-stimulatory effects such as CD8^+^ cytotoxic T lymphocyte responses. After receptor-mediated endocytosis by antigen-presenting cells (APC), peptide-loaded gp96 is presented on major histocompatibility complex class I molecules to T cells, which leads to APC activation and increased production of tumor necrosis factor (TNF) and IL-12 (5–7). Extracellular gp96 also induces TLR 4-dependent IL-12 production in dendritic cells (8). Type 1 cytokines, including interleukin (IL)-12 and IL-18, are known to play a key role in human IBD (9–14) and in murine intestinal inflammation (15–17) through their synergistic induction of IFNγ synthesis (18).

To elucidate the critical role of gp96 in promoting T-helper 1 (Th1)-mediated intestinal inflammation we applied two different models of murine inflammation. First, we injected mice daily with IL-12/IL-18, which led to murine intestinal inflammation (15, 19, 20). In order to make our preclinical study more generalizable, we chose TNBS (2,4,6-trinitrobenzenesulfonic acid) as our second model. TNBS represents a model of chemical induced intestinal inflammation and is commonly used to mimic a transmural Th-1 cell-dependent colitis, which causes epithelial injury of the colon (17) and has been widely used to study cytokine secretion patterns and effects of immunotherapies (21). We then tested blocking gp96 in both models of disease by injecting a gp96-blocking peptide (gp96-II).

As we have shown previously, gp96-II is a synthetic peptide that binds to and antagonizes gp96-mediated lipopolysaccharide (LPS)-induced cytokine production in freshly isolated human peripheral blood mononuclear cells (PBMCs) and in a murine LPS-induced endotoxin model (22). Others have shown that peptide-based inhibitors to gp96 can block the HSP90–LPS interaction (23) and that the gp96-II peptide inhibits endogenous gp96 in an allogeneic islet transplantation model by improving islet graft function (24).

Our study here shows that gp96-II peptide has actions beyond blockade of TLR pathways, notably by reducing IL-12/IL-18-, IL–1β- and anti-CD3-induced cytokine production *in vivo* and *in vitro*. Thus gp96 is a highly prospective target for the development of broad-spectrum therapies against multifactorial diseases such as IBD.

## Materials and Methods

### Reagents

RPMI 1640, phosphate-buffered saline (PBS, i.e., vehicle), fetal calf serum (FCS) and penicillin/streptomycin were purchased from Cellgro, Herndon, VA, USA. Pooled human serum was acquired from MP Biomedicals, Solon, OH, USA. LPS (O55:B5), anti-CD3 mAb and Ficoll Hypaque were from Sigma-Aldrich, St. Louis, MO, USA. Canine gp96 protein (Cat # G3057-41, US Biological, Swampscott, MA, USA). We purchased the detection kit for lactate dehydrogenase (LDH) from BioVision (Mountain View, CA, USA). *Staphylococcus epidermidis* (St. epi.) was obtained from the American Type Culture Collection (strain 49134), grown overnight in suspension cultures in LB medium (Difco, Detroit, MI, USA), centrifuged, washed in pyrogen-free vehicle and a small sample was removed for determination of number of organisms by pour plate cultures. The suspension was boiled for 30 min and then remained at room temperature for 24 h. The boiled suspension was diluted in pyrogen-free vehicle to 10 million organisms per milliliter and frozen in small aliquots at −70°C. Recombinant human IL–1β, human and murine IL-12 were obtained from Peprotech, Rocky Hill, NJ, USA, and human and murine IL-18 from MBL International, Woburn, MA, USA. The gp96-II peptide is not commercially available and was provided by Compugen, Tel-Aviv, Israel. Peptide designation residues: gp96-II (active) 444–480 LNVSRETLQQHKLLKVIRKKLVRKTLDMIKKIADDKY.

### Isolation of Splenocytes, PBMC and Cell Culture

Spleens of each mouse strain were aseptically removed, macerated and passed through a 70-µm cell strainer. Splenocytes were washed with PBS twice, centrifuged and re-suspended in RPMI with 5% FCS and cultured at 5 × 10^6^/ml in a 24-well flat bottom plate and stimulated as indicated. After 24 h, the supernatant medium was removed for measuring secreted cytokines.

The Colorado Multiple Institutional Review Board approved experiments involving human blood. After informed consent was obtained, PBMCs were isolated from peripheral venous blood of healthy volunteers by Ficoll Hypaque density gradient centrifugation. After isolation, cells were counted and examined for viability by trypan blue exclusion. For experiments on PBMC, these cells were used without further treatment. Thereafter, 0.5 × 10^6^ cells were resuspended in 0.3 ml fresh RPMI containing 1% human serum and primocin and plated into 48-well flat bottom polystyrene plates. Cells were then either stimulated or remained untreated as controls. After 1 day at 37°C and 5% CO_2_, supernatants were taken and the cells were lysed in lysis buffer (50 mM Tris, pH 7.4, 150 mM NaCl, 2 mM EDTA, 2 mM EGTA, 10% glycerol, 1% Triton X-100, 40 mM β-glycerophosphate, 50 mM sodium fluoride, 200 µM sodium vanadate, 10 µg/ml leupeptin, 10 µg/ml aprotinin, 1 µM pepstatin A and 1 mM phenylmethylsulfonyl fluoride) and frozen at −80°C. Before assay, the lysates were clarified by centrifugation at 20,000 × *g* for 10 min and the pellet discarded.

### Animal Studies

#### IL-12/IL-18-Induced Murine Intestinal Inflammation

Animals—8 to 12-week-old C57BL/6 mice were purchased from The Jackson Laboratory (Bar Harbor, ME, USA), housed five per cage and kept on a 12-h light–dark cycle. The University of Colorado Institutional Animal Care and Use Committee approved all experiments.

After daily intraperitoneal (i.p.) injection of male C57BL/6 (3 months, 20–21 g) with 1 μg/mouse of IL-12 and IL-18, mice exhibited chronic intestinal inflammation and a type 1 immune response, causing weight loss and increased serum cytokine levels similar to that described in Ref. (15, 20). For experiments, animals were injected with IL-12/IL-18 in combination with 60 μg/mouse gp96-II peptide (22) and a control group with vehicle. On day 5, mice were anesthetized by using the Open-Drop exposure to isoflurane and plasma was obtained by orbital bleeding into tubes which contained heparin. Animals were then humanely killed by cervical dislocation and spleens were harvested for splenocyte isolation. A small aliquot of the blood was taken for white blood cell counts.

#### Clinical Assessment of IL-12/IL-18 Induced Murine Intestinal Inflammation

Clinical assessment of IL-12/IL-18 induced intestinal inflammation was performed in all animals at day 2 after treatment, before the vehicle animals started to die at day 3. A combinational index of disease was determined by scoring (research staff blinded to treatment) the change in body weight (%), stool consistency and occult/gross blood stools (Table 1) (25). The Disease Activity Index (DAI) was then calculated as the average of the three values.

**Table 1:** Scoring system for Disease Activity Index (DAI).

Score	Weight loss (%)	Stool consistency	Occult/gross blood in stools
0	None	Normal stool	No blood
1	1–5	Slightly loose	Slightly bloody
2	6–10	Loose stool	Positive blood
3	>10	Diarrhea	Gross bleeding

*The degrees of body weight loss, stool consistency and occult/gross blood in stools were quantified as described. The DAI was calculated as the sum of the three scores (25)*.

*Stool consistency: normal stool, stool with an appearance of well-formed pellets; slightly loose stool, loose stool with pasty, semi-formed, soft materials that do not adhere to anal fur; diarrhea, liquid stool that adheres to anal fur. Gross bleeding, an appearance of visible blood adhering to anal fur*.

#### Induction of TNBS (2,4,6-Trinitrobenzenesulfonic Acid) Colitis in Mice

Eight week old C57BL/6 mice were obtained from the in-house breeding stock at the University of Mainz, Germany. The University of Mainz Animal Care and Use Committee approved all experiments. A total of 60 animals were allocated to 6 groups. The model employs the use of TNBS colitis by administering a total volume of 100 µl of 2 mg TNBS intrarectally at day 0. After day 1, animals were injected intraperitoneally once daily with either placebo or with corticosteroid (prednisolone: 1 g/mouse, which we used as a positive control in this setting) or with 60 µg/mouse of gp96-II peptide twice daily, over a period of 7 days and weight was monitored daily. On day 7, the animals were humanely killed by cervical dislocation.

### Whole Blood Assay

To investigate the long-term protective effects of gp96-II peptide, we performed *ex vivo* whole blood assays. Whole blood of animals from the IL-12/IL-18 ± gp96-II intestinal inflammation model was challenged *in vitro* with a “second hit” (i.e., stimulation with a TLR2-, TLR4-agonist or IL-1β) without further *in vitro* gp96-II peptide treatment. Blood was obtained by orbital bleeding after a 5-day injection series. A small aliquot was taken for white blood cell counts. The remaining whole blood was diluted 1:5 in RPMI and stimulated for 20 h. After this incubation, cultures were lysed with Triton X-100 (final concentration of 0.5%) and underwent a freeze–thaw cycle before cytokine determination.

### Electrochemiluminescence (ECL) Assays and Enzyme-linked Immunosorbent Assay (ELISA)

Human IL-1α, IL–1β, IL-6, IFNγ and TNF as well as murine IL-6, IL-1α, IL–1β and TNF were measured using specific antibody pairs and an Origen Analyzer (Wellstat Diagnostics, Gaithersburg, MD, USA) as described (26). Antibody pairs for all cytokines were obtained from R&D Systems with the exception of the purchase of IFNγ from Fitzgerald Industries International (Concord, MA, USA). Murine IFNγ was determined by ELISA (R&D Systems) according to the manufacturer’s instructions. Recombinant cytokines for ECL or ELISA standards were obtained from R&D Systems or Peprotech (Rocky Hill, NJ, USA).

### Cytokine Arrays

Equal volumes of cell culture supernatants or equal protein concentrations from lysates were incubated with the precoated Human Cytokine Antibody Array membranes (Proteome Profiler Arrays™, R&D Systems) according to the manufacturer’s instructions. Equal loading of protein was ascertained by densitometry of the positive control spots on the dot blot as well as by measuring total protein; differences in cell death were excluded by LDH measurements. Analysis was performed as previously described (26).

### Luminex Analysis: *In Vitro* Study of gp96 Peptide Variants

Peripheral blood mononuclear cells from three healthy human donors were incubated with nine gp96 peptide variants (30 µg/ml). After addition of 1 µg/ml of LPS for 24 h, or of anti-CD3 mAb (Sigma, St. Louis, MO, USA) for 48 h, the concentrations of several cytokines were measured in the supernatants using a Luminex analyzer (IS100, Luminex Corporation) and bead-based reagents (Upstate Biotechnology).

### Reverse Transcription Quantitative Real-time Polymerase Chain Reaction (RT-PCR)

After 4 h of LPS stimulation, PBMC cell cultures were spun down and supernatants were analyzed for LDH and human TNF. Total RNA containing miRNAs was extracted from the cell pellet according to the manufacturer’s instructions using the mirVana miRNA Isolation kit (Ambion, Austin TX, USA). Primer pair: 18S RNA: Hs99999901_s1 and hTNF Hs99999043_m1 (Applied Biosystems, Foster City, CA, USA).

For mRNA quantification, cDNA was synthesized from isolated RNA using a High Capacity cDNA Reverse Transcription kit (Invitrogen) according to the manufacturer’s instructions. Reverse transcription quantitative real-time polymerase chain reaction (RT-qPCR) was performed using an Applied Biosystems 7900HT Fast Real-Time PCR System. Human TNF DNA levels were determined by RT-qPCR with the Taqman^®^ Gene Expression Master Mix by Applied Biosystems (Applied Biosystems, Foster City, CA USA). Fold changes in expression were calculated by the 2^−ΔΔ^*^C^*^q^ (*C*_q_ = quantification cycle) using the RQ Manager 1.2 and 18S FAM as reference gene. The primer/probe sets targeted human TNF. Total DNA content was quantified with a NanoDrop (Thermo Fisher Scientific); each sample was measured in triplicate.

### Histology

The distal colon was taken at necroscopy on day 7, processed, embedded and stained with H&E as previously described in Ref. (21). An experienced gastroenterologist blinded to the treatment performed scoring. A combined score ranging from 0 to 6 was used to quantify colitis. Inflammatory cell infiltration was scored from 0 to 3 and tissue damage was scored from 0 to 3. Absence or occasional inflammatory infiltrate in the lamina propria was scored as 0, increased numbers of inflammatory cells restricted to the lamina propria was scored as 1, inflammatory infiltrates reaching the submucosa was scored as 2 and transmural cell infiltration was scored as 3. The subscore for tissue damage took into account epithelial lesions with no mucosal damage scored as 0, focal crypt lesions scored as 1, surface mucosal erosions or focal ulceration scored as 2 and extensive mucosal damage affecting the submucosa was scored as 3. The combined inflammatory and histological score resulted in the overall score ranging from 0 (no changes) to 6 (severe inflammatory infiltrate and mucosal damage).

### Statistical Analysis

Data sets (raw data) were first tested for normality and equal variance (*P* value to reject = 0.05) with Sigma Plot 12.5 (Systat software). Thereafter, the appropriate statistical test was applied, which included paired or unpaired Student’s *t*-test and/or by the Mann–Whitney rank sum or the Wilcoxon signed rank tests on raw data and one-way analysis of variance (ANOVA) or one-way ANOVA on ranks. All data that underwent statistical analysis are presented either as means of absolute cytokine concentrations, means of normalized cytokine concentrations, or means of percent change ± SEM.

### Ethical Considerations

This study and protocol were carried out in accordance with the recommendations and approval by the Colorado Multiple Institutional Human Review Board. All subjects gave written informed consent in accordance with the Declaration of Helsinki. The animal studies and protocols were carried out in accordance with the recommendations of the Animal Review Board of the University of Colorado Denver and the Animal Review Board of the University of Mainz.

## Results

### gp96-II Peptide Is Protective in Two Murine Models of Intestinal Disease

**Figure 1:**
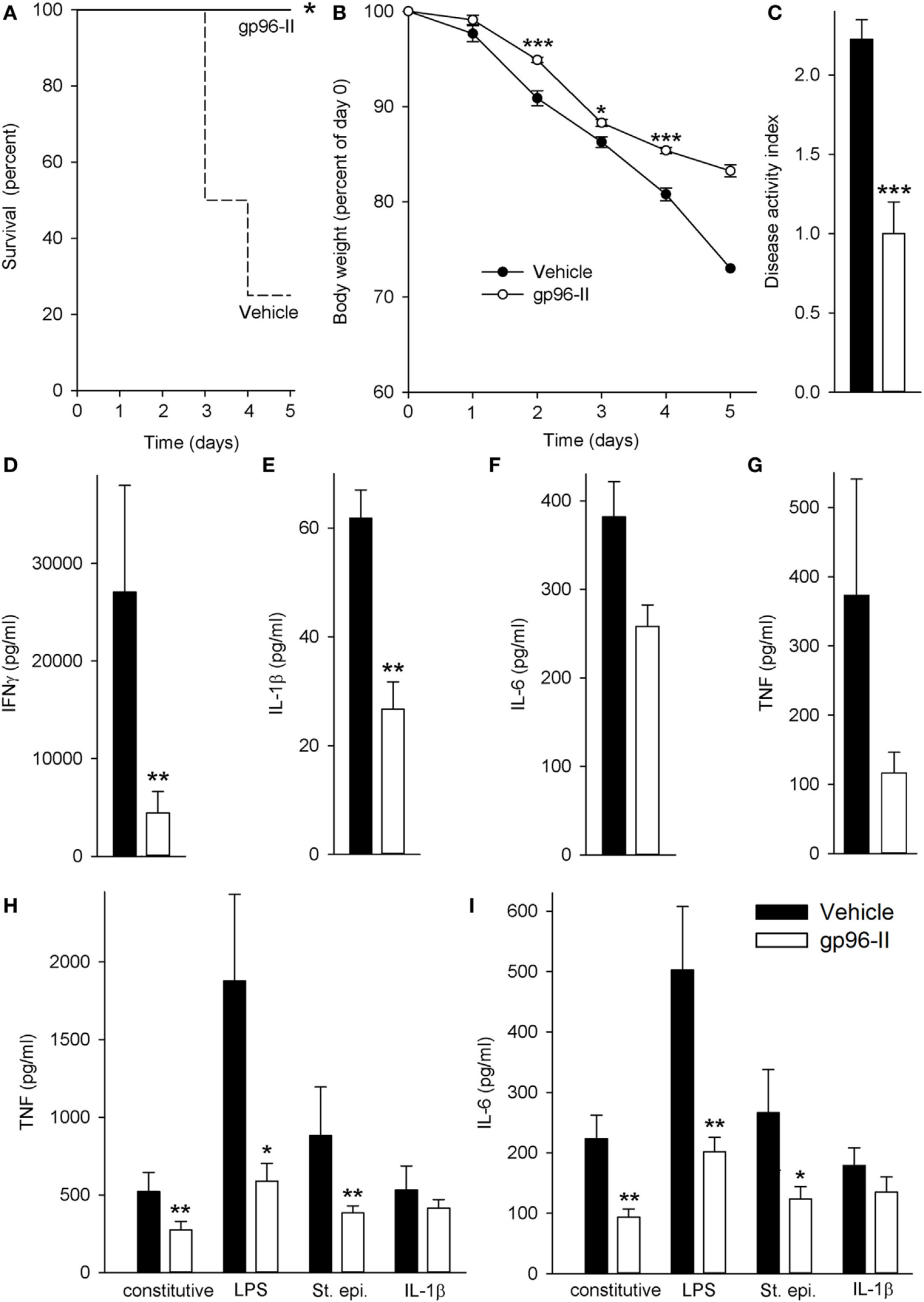
Amelioration of IL-12/IL-18-induced intestinal inflammation by gp96-II peptide *in vivo*. C57BL/6 mice were injected daily intraperitoneally with either vehicle or gp96-II peptide (60 µg) and with IL-12 (1 µg) and IL-18 (1 µg). **(A)** Kaplan–Meier analysis of survival of vehicle-treated mice (dashed line) vs. gp96-II peptide-injected mice (solid line). *n* = 10 mice per group; *P < 0.05 for vehicle vs. gp96-II peptide. **(B)** Means of percent changes in body weight ± SEM of surviving mice injected with IL-12/IL-18 + vehicle (filled symbols) or IL–12/IL–18 + gp96-II peptide (open symbols). *n* = 10 mice per group, identical animals to those in panel **(A)**; *P < 0.05 and ***P < 0.001 for gp96-II peptide vs. vehicle. No statistics on last data point as only two survivors in the vehicle group. **(C)** Disease Activity Index analysis, IL-12/IL-18 + vehicle-injected mice (filled bars) and in animals treated with IL–12/IL-18 + gp96-II peptide (open bars) on day 2. *n* = 9–10 mice per group; ***P < 0.001 for gp96-II peptide vs. vehicle. **(D–G)** Concentrations of plasma IFNγ, IL-1β, IL-6 and tumor necrosis factor (TNF) ± SEM in IL-12/IL-18 + vehicle injected mice (filled bars) and in animals treated with IL–12/IL-18 + gp96-II peptide (open bars) on day 5. *n* = 6 mice per group; **P < 0.01 for vehicle vs. gp96-II peptide treatment. **(H,I)** Comparison of cytokine abundance in *ex vivo* whole blood assays from mice that had received daily injections with IL–12/IL-18 + vehicle (filled bars) or IL-12/IL-18 + gp96-II peptide (open bars) for 5 days. Whole blood was then challenged *in vitro* for 24 h with lipopolysaccharide (1 µg/ml), *Staphylococcus epidermidis* (1:1,000), or IL-1β (25 ng/ml). The graphs show means of cytokine concentrations ± SEM; *n* = 6; *P < 0.05 and **P < 0.01 for vehicle vs. gp96-II peptide, absence of a symbol indicates a non-significant difference.

Daily injection with IL-12 and IL-18 led to severe acute intestinal inflammation, thereby resulting in symptoms such as bloody diarrhea and fever. IL-12/IL-18-treated mice received either a daily dose of gp96-II peptide or were injected with identical volumes of vehicle. Figure 1A demonstrates the protection afforded by gp96-II peptide against the IL-12/IL-18-triggered intestinal inflammation. After 5 days, 80% of the vehicle-injected mice had died, whereas in the gp96-II peptide-treated group, all mice survived. gp96-II peptide also markedly attenuated the IL-12/IL-18-induced weight loss (Figure 1B) and reduced the severity of diarrhea compared with the control group (data not shown). To determine the clinical presentation of IL-12/IL-18-induced intestinal inflammation on all animals, an assessment was performed on day 2, just before vehicle-treated control animals began to perish. The vehicle group presented with diarrhea and bloody stools, which were infrequent or not evident in the gp-96-II-treated group. No gross bleeding was observed in any of the animal groups. The clinical assessment DAI of intestinal inflammation severity was found to be significantly lower in the gp96-II-treated animals when compared to vehicle-injected mice (Figure 1C; Table S1 in Supplementary Material).

Next, we investigated plasma cytokine abundance in the surviving mice on day 5. As shown in Figure 1D, daily gp96-II peptide injections reduced IL-12/IL-18-induced plasma IFNγ by 89% compared to controls and inhibited plasma cytokine levels of IL-1β by 63%, IL-6 by 43% and TNF by 70% (Figures 1E–G). White blood cell counts showed no significant difference between the groups.

Having thus demonstrated that gp96-II peptide is a potent inhibitor of IL-12/IL-18-induced pro-inflammatory cytokines *in vivo*, we questioned whether residual gp96-II peptide afforded extended protection by performing *ex vivo* whole blood assays. We studied surviving mice that had either received daily injections with IL–12/IL-18 + vehicle or IL-12/IL-18 + gp96-II peptide. The mice were bled at day 5 and whole blood was challenged immediately *in vitro* for 24 h with TLR4-agonist LPS, TLR2-agonist St. epi. or the pro-inflammatory cytokine IL-1β, without any addition of gp96-II peptide to the cultures. As shown in Figures 1H,I, residual gp96-II peptide from the *in vivo* injections inhibited constitutive expression of TNF by 48% and IL-6 by 58%. Comparing whole blood from gp96-II and vehicle-treated mice; we found TNF was reduced by 69% when the second *in vitro* hit was LPS, 56% when it was St. epi. and 22% when it was IL-1β. Likewise, IL-6 expression was 60, 53 and 25% lower in response to all “second hit” conditions.

**Figure 2:**
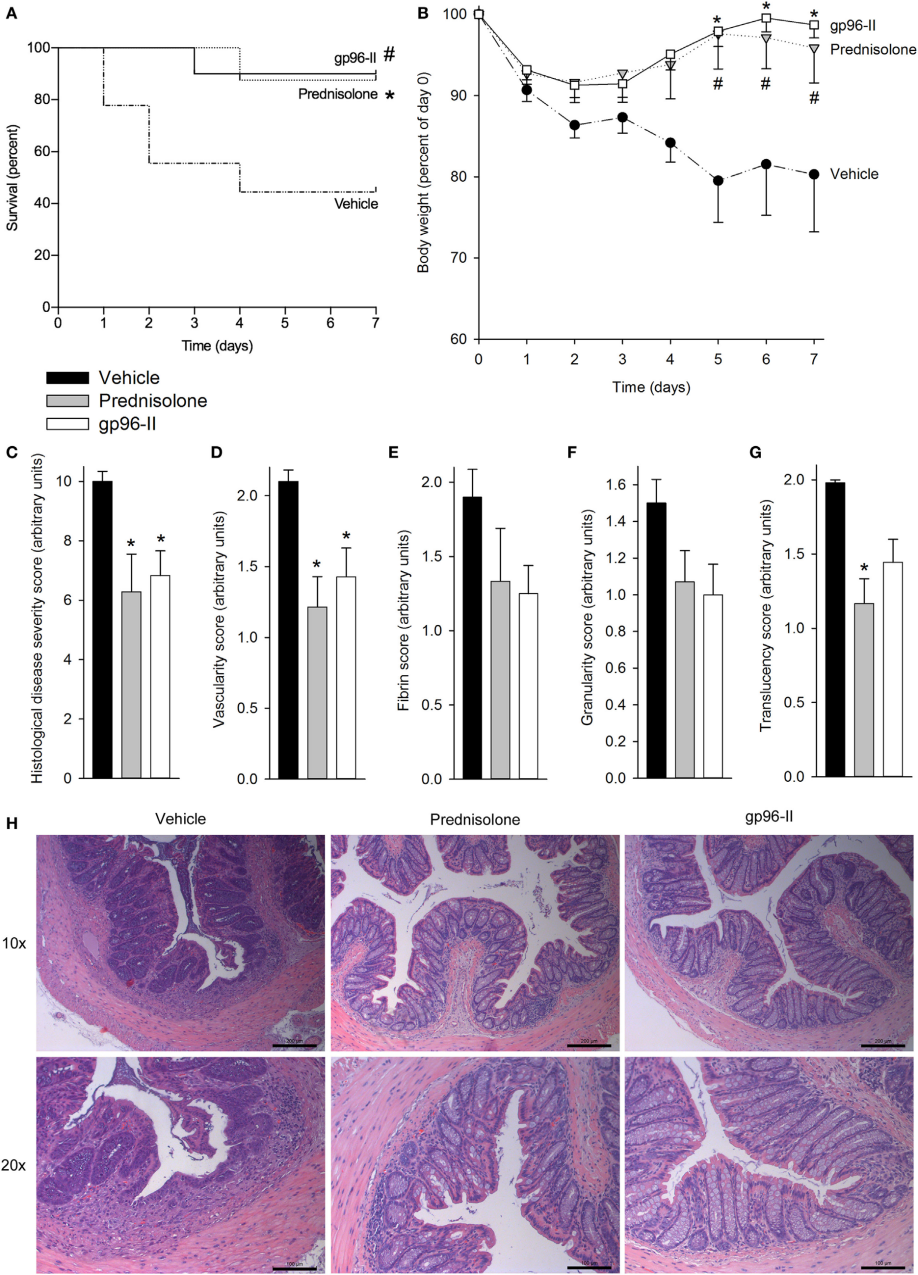
Amelioration of TNBS-induced colitis by gp96-II peptide. Colitis was induced by intrarectal application of TNBS on day 0. Male C57BL/6 mice either received twice daily intraperitoneal injections of vehicle or gp96-II peptide (60 µg), or were administered prednisolone (1 g) once daily for 7 days. **(A)** Kaplan–Meier analysis of survival of vehicle-treated mice (dashed line) vs. gp96-II peptide-injected mice (solid line) or vs. prednisolone injected mice (dotted line). *n* = 10 mice per group; ^#^*P* < 0.05 for vehicle vs. gp96-II peptide, *P < 0.05 for vehicle vs. prednisolone. **(B)** Bodyweight in percent of day 0 ± SEM is shown for the gp96-II peptide (open squares) (*n* = 10), prednisolone (gray triangles) (*n* = 8) and vehicle (solid circles) groups (*n* = 9); *P < 0.05 for gp96-II peptide vs. vehicle; ^#^*P* < 0.05 for prednisolone versus vehicle. **(C)** Postmortem histological disease severity scores of the colons (arbitrary units; see Materials and Methods) on day 7 ± SEM. **(D–G)** Endoscopy was performed on anesthetized mice on day 3 of the experiment and vascularity, fibrin, granularity and translucency scores (arbitrary units ± SEM) were obtained. *n* = 7 in the vehicle (filled bars) and prednisolone (gray bars) groups, *n* = 9 in the gp96-II peptide group (open bars); *P < 0.05 for gp96-II peptide or prednisolone vs. vehicle, absence of a symbol indicates a non-significant difference. **(H)** Intestinal sections were H&E stained and analyzed on day 7, *n* = 7–9 per group. One representative image per treatment group depicting the colon at a low (10×) and high (20×) magnification. Scale bars: 200 µm for 10× magnification and 100 μm for 20× magnification.

Our second model inducing colitis caused by intrarectal instillation of TNBS in 50% ethanol triggered mucosal inflammation mediated by a Th1 response with excessive pro-inflammatory cytokine production (17). The mice were also treated once daily with vehicle as a control, with 1 g of the corticosteroid prednisolone or twice daily with 60 µg of gp96-II peptide. The vehicle-treated group exhibited the highest mortality (Figure 2A) and most pronounced weight loss (Figure 2B); both prednisolone and gp96-II peptide completely protected the mice from the deleterious effects of TNBS (Figures 2A,B).

To assess tissue damage, the distal colon of each animal was obtained at necroscopy and sections were H&E stained and scored, with the assessor blinded to treatment. Prednisolone and gp96-II peptide were equally potent in reducing histological disease severity scores by 40 and 35%, respectively (Figure 2C).

Endoscopy on anesthetized animals on day 3 of the experiment (27) showed that mice treated with gp96-II peptide or with prednisolone had improved endoscopic scores compared with vehicle-treated control mice: vascularity, fibrin, granularity and translucency scores were reduced by up to 49% by prednisolone and by up to 30% by gp96-II peptide (Figures 2D–G). As shown in Figure 2H, mice exposed to TNBS displayed changes in intestinal morphology as characterized by edema and infiltration by inflammatory cells in the mucosal and sub-mucosal layers and tissue damage. Daily injections of gp96-II peptide or prednisolone almost completely prevented these morphological changes as shown by significantly ameliorated histological disease severity scores (Figure 1C).

### gp96-II Peptide Reduces Cytokines in Freshly Isolated Murine Splenocytes

**Figure 3:**
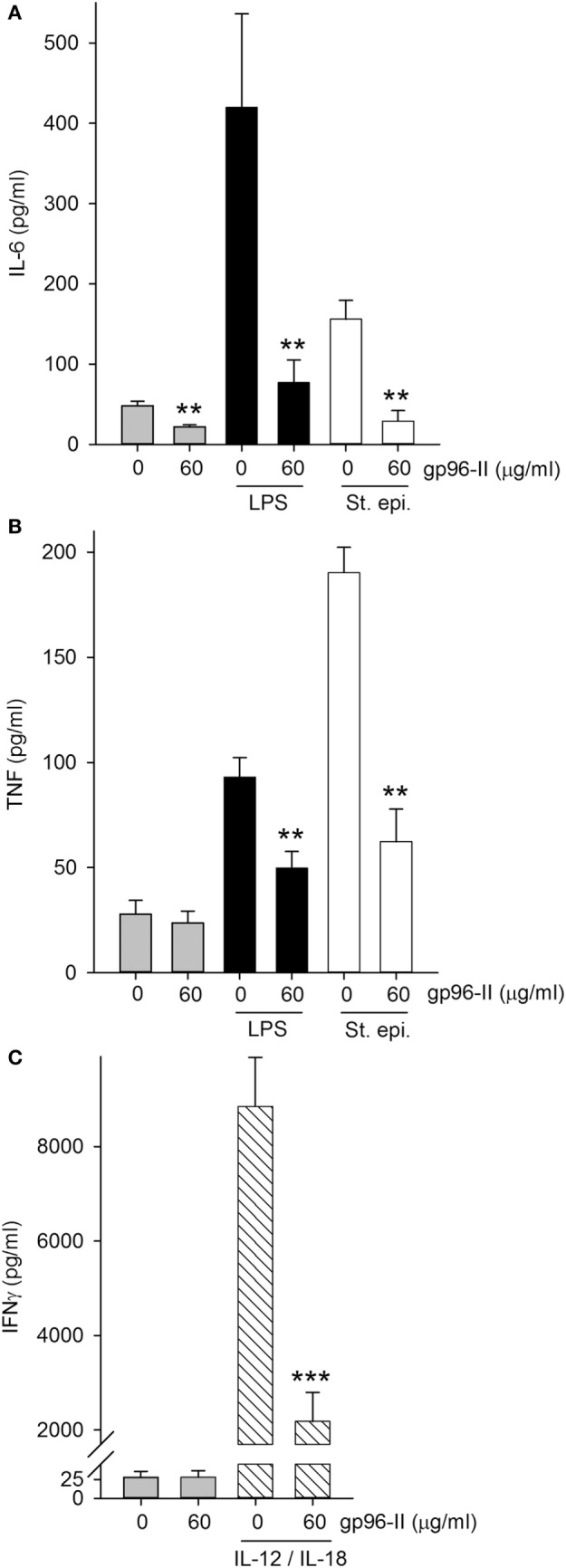
Effect of gp96-II peptide on TLR- or IL-12/IL-18-induced cytokine production in murine splenocytes. **(A,B)** Cells were incubated for 24 h with vehicle, lipopolysaccharide (LPS) (100 ng/ml), or *Staphylococcus epidermidis* (St. epi.) (1:4,000) in the presence or absence of gp96-II peptide (60 µg/ml). The graphs display means of absolute cytokine abundance ± SEM. **(C)** IFNγ protein abundance in cells stimulated for 24 h with either vehicle or IL-12 (20 ng/ml) + IL-18 (50 ng/ml) in the presence or absence of 60 µg/ml of gp96-II peptide. *n* = 6; **P < 0.01 and ***P < 0.001 for LPS, St. epi. or IL-12/IL-18 alone vs. stimulus + gp96-II peptide.

gp96-II peptide had an inhibitory effect in freshly isolated murine splenocytes as shown by its action in markedly reducing constitutive IL-6 by 54%, LPS-induced TNF and IL-6 by 48 and 81% respectively and St. epi.-induced TNF and IL-6 by 67 and 81% respectively (Figures 3A,B). gp96-II peptide inhibited IL-12 + IL-18-induced IFNγ secretion by 75% at a concentration of 60 µg/ml (Figure 3C).

### Inhibition of LPS-Induced *TNF* mRNA by gp96-II in PBMC Does Not Affect Cell Viability

**Figure 4:**
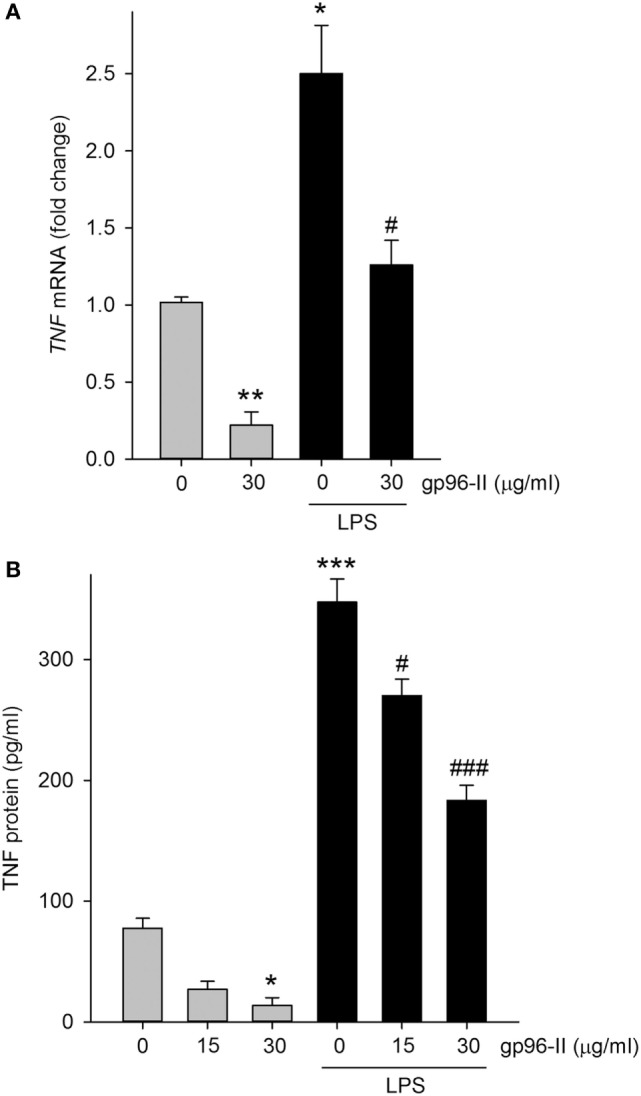
Tumor necrosis factor (*TNF*) mRNA and protein in lipopolysaccharide (LPS)-stimulated peripheral blood mononuclear cells with and without gp96-II peptide. Cells were incubated with or without LPS in combination with gp96-II peptide (30 µg/ml) for 4 h. **(A)** Fold change in *TNF* mRNA ± SEM; **(B)** TNF protein abundance ± SEM. *n* = 5; *P < 0.05; **P < 0.01; and ***P < 0.001 for control vs. stimulation with LPS or gp96-II peptide alone; ^#^*P* < 0.05 and ^###^*P* < 0.001 for LPS plus gp96-II peptide vs. LPS alone.

Treatment of PBMC with 30 µg/ml gp96-II peptide decreased *TNF* mRNA expression at 4 h, under steady-state conditions by 80% and after LPS stimulation by 52% (Figure 4A). Accordingly, protein abundance of LPS-induced TNF was also decreased (Figure 4B) by up to 53%. Ruling out the possibility that the anti-inflammatory effects of gp96-II peptide were attributable to cell death, we found no increase in LDH in the culture supernatants of PBMC treated with 15, 30, 60 and 120 µg/ml of gp96-II peptide for 4 h compared to vehicle; in fact, gp96-II peptide negated the small increase in LDH induced by LPS in PBMC (not shown). We also performed flow cytometry to investigate the effect of gp96-II peptide on specific subpopulations of PBMC. In cells treated with vehicle, LPS alone or LPS + gp96-II peptide (30 µg/ml) for 20 h, we found no differences in overall viability, in the percentage of lymphocytes and macrophages among live cells or in the percentage of CD3^+^ T-cells and CD19^+^ B-cells among lymphocytes (not shown).

### Anti-inflammatory Activity Screening of Nine gp96 Peptide Variants

To determine the best anti-inflammatory gp96 peptide variant, we tested nine different gp96 peptide variants, termed gp96-I to -IX, in PBMC at a concentration of 30 µM. As shown in Figures S1A,B in Supplementary Material, peptide gp96-II (CGEN-25007) reduced LPS-induced IL-6 by 91%, IL-8 by 93%, MIP-1α by 73%, IL-1β by 93% and TNF by 60% compared to the other peptides and even outperformed the inhibitory effects of dexamethasone. Reduction of anti-CD3-induced cytokine production by gp96-II peptide was comparable for IL-1β, IL-12p40, IL-12p70 and TNF, whereas IL-1α, GM-CSF and IL-2 were not affected (Figures S1C,D in Supplementary Material). These data confirm that gp96-II peptide (CGEN-25007) is the peptide with most potent anti-inflammatory activity in LPS- as well as anti-CD3-stimulated PBMC.

### gp96-II Peptide Abrogates Production of Pro-inflammatory Cytokines in Human PBMC

**Figure 5:**
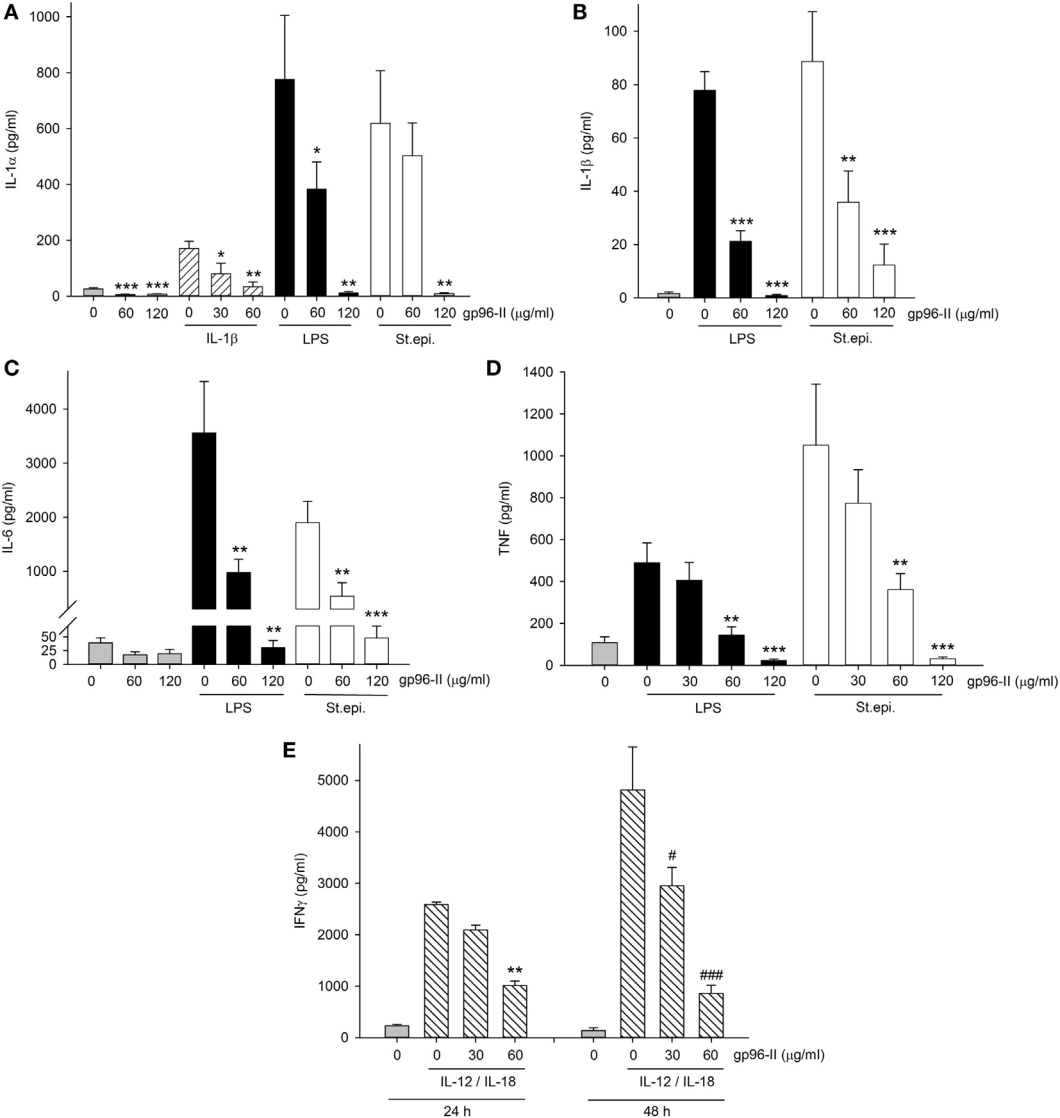
Effect of gp96-II peptide on TLR- and cytokine-induced cytokine production in human peripheral blood mononuclear cells (PBMC). **(A–D)** Cells were incubated with lipopolysaccharide (LPS) (100 ng/ml), IL-1β (10 ng/ml), *Staphylococcus epidermidis* (St. epi.) (1:4,000) or vehicle, alone or in combination with gp96-II peptide for 24 h. The concentrations of gp96-II peptide in micrograms per milliliter are indicated. Lysates **(A)** or supernatants **(B–D)** were analyzed for cytokine abundance. The panels depict means of absolute concentrations of cytokines ± SEM, *n* = 4; *P < 0.05; **P < 0.01; ***P < 0.001 for IL-1β-, LPS-, or St. epi.-stimulated cells + gp96-II peptide vs. stimulated cells alone. **(E)** PBMC from four donors were incubated for the indicated periods of time in the presence or absence of IL-12 (20 ng/ml) + IL-18 (25 ng/ml) with or without gp96-II peptide at the indicated concentrations in micrograms per milliliter. **P < 0.01 for IL-12/IL-18 vs. IL-12/IL-18 + gp96-II peptide at 24 h; ^#^*P* < 0.05 and ^###^*P* < 0.001 for IL-12/IL-18 vs. IL-12/IL-18 + gp96-II peptide at 48 h. Absence of a symbol indicates a non-significant difference.

To investigate the clinical relevance of gp96-II peptide on inhibition of TLR2/4- or IL-1b-triggered inflammatory responses in primary human cells, we isolated fresh PBMCs and stimulated them with gp96-II peptide, either alone or in combination with LPS, or with heat-killed St. epi. or IL-1β. We found that gp96-II peptide effectively blocked the production of pro-inflammatory cytokines in PBMC. For example, 60 and 120 µg/ml gp96-II peptide decreased the concentrations of LPS-induced IL-1α (by 50 and 98%, respectively), IL–1β (by 72 and 99%, respectively), IL-6 (by 70 and 94%, respectively) and TNF (by 70 and 94%, respectively) compared with LPS alone (Figures 5A–D). Inhibition of the induction of pro-inflammatory cytokines by St. epi. was similar.

We also tested IL-1β as a pro-inflammatory stimulus and observed that 30 µg/ml of gp96-II peptide was sufficient to inhibit production of IL-1α (Figure 5A), an unexpected result as 30 µg/ml did not reduce TLR-induced cytokines (Figure 5D). However, IL-1β-induced IL-6 and TNF remained unaffected by 30 and 60 µg/ml of gp96-II peptide. In addition, we investigated the effect of gp96-II peptide on IL-12/IL-18-induced IFNγ in human PBMC (Figure 5E). gp96-II peptide inhibited IL-12/IL-18-induced IFNγ secretion in human PBMC at a concentration of 30 µg/ml (by 20%) and at 60 µg/ml (by 61%) after 24 h and by 82% after 48 h (Figure 5E). Compared to TLR ligands, induction of IL-1β, IL-6, IL-1α and TNF was moderate; however, the magnitude of the reduction in IL-1α by gp96-II peptide at 60 µg/ml was 50% (not shown). To rule out unspecific effects of the gp96-II peptide, we showed that mutated nonsense peptides had no effect on any of the parameters we assessed (not shown).

**Figure 6:**
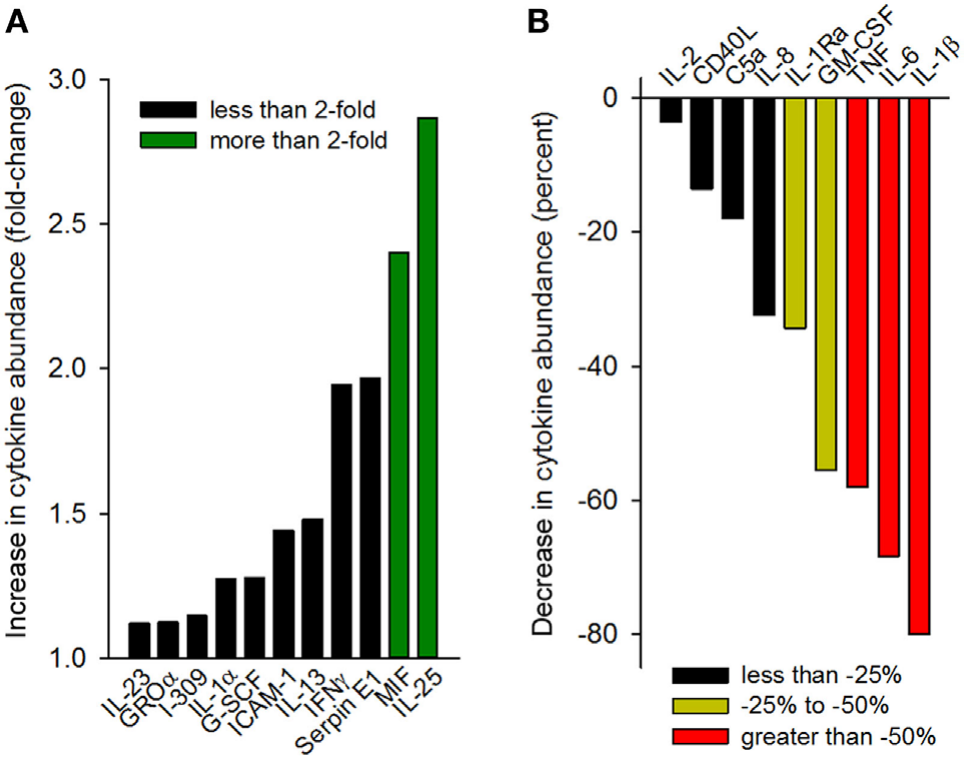
Cytokine array analysis of supernatants of peripheral blood mononuclear cell cultures (two donors) treated with 100 ng/ml lipopolysaccharide with or without gp96-II peptide (30 µg/ml) for 24 h. Densitometric analysis of the blots is depicted as normalized density per square millimeter. Panel **(A)** describes the increase in cytokine abundance (fold change) and panel **(B)** describes the decrease in cytokine abundance in percent.

Having observed that gp96-II peptide was highly effective in reducing TNF, IL-1β, IL-6 and IL-1α, we further investigated the effect of gp96-II peptide in LPS-stimulated PBMC using an array of 40 human mediators of inflammation. Figure 6A shows the increase in cytokine abundance and Figure 6B shows the decrease in cytokine abundance. In addition to confirming the results described above, the array revealed decreases in the abundance of GM-CSF and IL-1Ra, as well as increases in RANTES (threefold), IL-25 and MIF (twofold to threefold), whereas IL-10, IL-4 and IL-5 were measured but were undetectable in all conditions.

## Discussion

Inflammatory bowel disease, which encompasses UC and CD, is a chronic disorder characterized by severe intestinal inflammation. Our aim was to advance the prospects for anti-inflammatory agents by focusing on the role of the DAMP molecule gp96 in IBD. The data we present here provide new mechanistic insights into the role of gp96 in IBD. Our major findings are that the synthetic molecule gp96-II peptide binds to and thereby blocks the pro-inflammatory activities of HSP gp96 *via* inhibition of TLR signaling and amelioration of intestinal inflammation. Furthermore, our study shows that the inhibitory functions of gp96-II peptide protect against cytokine-induced inflammation in PBMC from healthy human donors and primary murine cells. Our detailed analysis of gp96-II peptide provides new insights into the broad mechanistic functions of gp96 and highlights the value of gp96-II peptide as a starting point in the design of small molecule inhibitors for inflammatory diseases of the intestine.

Before performing any *in vivo* testing in murine models of intestinal disease, we first screened nine different gp96-IIs *in vitro* to assess their inhibitory properties. We found that the peptide gp96-II provided the most potent and versatile inhibition. Thus, we demonstrated that inflammation induced by T cell activation *via* CD3 ligation in PBMC is reduced with gp96-II peptide to a similar degree compared with its inhibitory effects on LPS-induced inflammation. gp96-II peptide thus acts as a multifunctional immune blocking agent, adding to earlier evidence that gp96-II peptide inhibits TLR2- and TLR4-induced pro-inflammatory (27) response in PBMC and murine splenocytes (22).

Our efforts in this study were directed at a more detailed analysis of the inhibitory effects of gp96-II peptide on pro-inflammatory cytokines and the associated immunobiology. The choice of IL-12/IL-18 for the creation of an inflammatory condition to model IBD was based on several studies. Among the pro-inflammatory cytokines, the IL-1 family member IL-18 plays an important role in a range of immune-mediated pathologies. For example, in 1999, three groups independently reported the pathological role of IL-18 in IBD patients (10–12). Subsequent studies confirmed that IL-18 has a role in the pathogenesis of human IBD as well as in murine intestinal inflammation (15, 16, 28–30). The high level of IL-18 that is generated in the inflamed gut causes further damage of the intestinal epithelium, thereby promoting penetration of gut bacteria across the compromised mucosa and later infiltration of T cells (1). Subsequently, alarmins and DAMP factors such as gp96 are released.

Extracellular gp96 then activates TLR2 and TLR4 and increases synthesis of pro-inflammatory cytokines (8), including vigorous IFNγ production by T cells, profound inflammatory responses and IBD pathology (9). In addition, gp96 is overexpressed in ileal epithelial cells in biopsies from patients with CD and promotes mucosal invasion of bacteria such as *Escherichia coli* (31). Tissues from CD patients are known to have elevated IL-12 transcripts and their lamina propria mononuclear cells produce higher levels of IL-12 compared to healthy subjects (13). Moreover, gp96 activates dendritic cells *via* the TLR2 and TLR4 pathways (8), and dendritic cells and macrophages produce more IL-12 in patients with CD, leading to a prototypical activation of APC and a shift toward Th1 differentiation (32). These findings support a strong pathological association of gp96, IL-12 and IL-18 in inflammatory diseases of the gut and highlights the importance of gp96 as a key-signaling player in intestinal type 1 immune responses.

Another important function exerted by IL-12 and IL-18 in synergy is a potent induction of IFNγ *in vivo* and *in vitro* (15, 19, 20, 33, 34), especially in IBD. One particular focus of our study was therefore to investigate the inhibitory effect of gp96-II on IFNγ, as it has previously been shown that gp96 protein expression is dose- and time dependently increased *in vitro* by IFNγ in lymphoid and epithelial cancer cells (7, 35). We show that inhibition of gp96 with gp96-II peptide significantly inhibits IL-12/IL-18-induced INFγ induction *in vitro* in PBMC as well as in murine splenocytes. To confirm the pivotal inhibitory effect of gp96-II peptide on IL-12/IL-18-induced inflammation *in vivo*, we utilized a chronic intestinal inflammation model in which a daily i.p. co-administration of IL-18 and IL-12 induces a severe systemic toxicity and type 1 immune response in mice, causing weight loss and increased serum cytokine levels (15, 19, 20). Strikingly, animals treated with daily i.p. gp96-II peptide injections were exempt from all severe symptoms induced by daily IL-12/IL-18 injections and also suffered significantly less from systemic inflammation than control mice.

We investigated the longevity of the protective effects of gp96-II peptide *via ex vivo* whole blood experiments. Our data presented here show that cells obtained from mice injected daily with IL-12/IL-18 and treated *in vivo* with gp96-II peptide exhibited a reduced pro-inflammatory cytokine expression when exposed to a “second hit” involving stimulation with a TLR2- or TLR4-agonist or IL-1β when compared to vehicle-treated animals. Collectively, these findings suggest that gp96 has a prominent role in cytokine regulation of intestinal inflammation and that gp96-II peptide strongly and lastingly inhibits key cytokines such as IL-1β, IFNγ, TNF and IL-6, thereby improving the outcome of murine intestinal inflammation (36).

To complement these investigations, we chose TNBS as our second model of murine intestinal inflammation, as the inflammation induced by TNBS is T cell driven and associated with elevated IFNγ, which causes epithelial injury of the colon. This model has been widely used to study cytokine secretion patterns and the effect of immunotherapies (17, 21). Elevated IFNγ production is an invariable feature of IBD pathogenesis, as reviewed in detail in Ref. (37). In this context, it is relevant that TNBS colitis can be treated with antibodies to IL-12, one of the key inducers of IFNγ (17). We therefore considered that TNBS-induced colitis is a model that is well-suited for testing the impact of continuous treatment with gp96-II peptide. Our study shows that TNBS-challenged mice, if treated with gp96-II, benefit from reduced weight loss, improved endoscopic scores and present with fewer morphological changes in the colon. Indeed, our data show that the inhibitor gp96-II peptide has anti-inflammatory properties as strong as those of prednisolone, which we used as a positive control in this setting. Clinically, corticosteroids are one of the mainstay therapies for IBD in human patients.

We can speculate that gp96-II peptide exerts its mechanistic effects by reducing the pro-inflammatory cytokines IL-1β, IL-6, TNF, and IFNγ in murine samples and in PBMC induced by different triggers. Interestingly the type 2-polarizing cytokine IL-25 was increased in PBMC, whereas IL-23, G-CSF, ICAM-1, IL-13, IL-2, CD40L, C5a and IL-8 showed little or no change. Thus, gp96-II may act *via* induction of IL-25, which is well known to have protective effects in IBD (25). In this context, we note that IL-25 inhibits IL-12 and Th-1 cell-driven inflammation in colonic samples from patients and experimental colitis (38).

Several attempts have been made to introduce cytokine-based therapies for patients with IBD. However, to date, anti-inflammatory cytokines such as IFNβ (34), IL-10 (39–41) and IL-11 (42) have failed to show benefit in patients with CD. A trial with a neutralizing antibody against IFNγ (fontolizumab) was stopped despite showing some beneficial effect (43), while anti-IL-17A (secukinumab) aggravated CD (44). Initial results with the anti-TNF antibody infliximab in patients with CD (45, 46) and other anti-TNF therapies such as adalimumab and certolizumab, pointed to improved outcome of many patients with IBD (CD and UC), but within 1 year only a third maintained remission (47).

Our study was motivated by the clear need for novel targets and therapies for human intestinal inflammatory disease. That gp96 has multiple and potent pro-inflammatory effects is already well documented, including dendritic cell activation, type 1 immune polarization and downregulation of its own receptors in a negative feedback loop (5). These actions, in combination with the therapeutic benefits we report on its synthetic inhibitor gp96-II peptide, makes gp96 a promising target for the development of therapies to restore intestinal homeostasis in IBD.

## Ethics Statement

This study and protocol were carried out in accordance with the recommendations and approval by the Colorado Multiple Institutional Human Review Board. All subjects gave written informed consent in accordance with the Declaration of Helsinki. The animal studies and protocols were carried out in accordance with the recommendations of the Animal Review Board of the University of Colorado Denver and The University of Mainz Animal Care and Use Committee.

## Author Contributions

All authors were involved in drafting the article or revising it critically for important intellectual content, and all authors approved the final version to be published. CAD and CAN-P had full access to all of the data in the study and take responsibility for the integrity of the data and the accuracy of the data analysis. Study conception and design: CAD, CAN-P, CB, SW, and MN. Acquisition of data: CAN-P, MFN, OL, YK, AO, IB, CB, SW, and MN. Analysis and interpretation of data: CAN-P, MFN, OL, YK, AO, IB, MKS, CB, SW and MN.

## Conflict of Interest Statement

The authors declare that the research was conducted in the absence of any commercial of financial interest that could be considered as a potential conflict of interest. Gp96-II was an in kind contribution from Compugen Ltd., 26 Harokmim Street, Holon 5885800, Israel.

## Supplementary Material

Figure S1 Anti-inflammatory activity of nine gp96-II peptides. gp96-II peptide (30 µM, open symbols) inhibits the production of cytokines from human peripheral blood mononuclear cells in response to a 24-h treatment with 1 µg/ml lipopolysaccharide (LPS) **(A,B)** or 48 h with 30 µg/ml anti-CD3 **(C,D)** in comparison to peptides gp96-I and III-X (30 µM, solid symbols) and dexamethasone. Individual cytokine abundance is depicted with each symbol representing one biological replicate. Horizontal lines show means.

Table S1 Stool consistency: normal stool, stool with an appearance of well-formed pellets; slightly loose stool, loose stool with pasty, semi-formed, soft materials that do not adhere to anal fur; diarrhea, liquid stool that adheres to anal fur. Gross bleeding, an appearance of visible blood adhering to anal fur.

## References

[1] Marsal J Agace WW. Targeting T-cell migration in inflammatory bowel disease. J Intern Med (2012) 272(5):411–29. 10.1111/j.1365-2796.2012.02588.x 22946654

[2] Multhoff G Heat shock proteins in immunity. Handb Exp Pharmacol (2006) 172:279–304. 10.1007/3-540-29717-0_12 16610364

[3] Yang Y Liu B Dai J Srivastava PK Zammit DJ Lefrancois L Heat shock protein gp96 is a master chaperone for toll-like receptors and is important in the innate function of macrophages. Immunity (2007) 26(2):215–26. 10.1016/j.immuni.2006.12.005 17275357 PMC2847270

[4] Staron M Yang Y Liu B Li J Shen Y Zuniga-Pflucker JC gp96, an endoplasmic reticulum master chaperone for integrins and toll-like receptors, selectively regulates early T and B lymphopoiesis. Blood (2010) 115(12):2380–90. 10.1182/blood-2009-07-233031 19965672 PMC2845896

[5] Singh-Jasuja H Scherer HU Hilf N Arnold-Schild D Rammensee HG Toes RE The heat shock protein gp96 induces maturation of dendritic cells and down-regulation of its receptor. Eur J Immunol (2000) 30(8):2211–5. 10.1002/1521-4141(2000)30:8<2211::AID-IMMU2211>3.0.CO;2-0 10940912

[6] Singh-Jasuja H Toes RE Spee P Munz C Hilf N Schoenberger SP Cross-presentation of glycoprotein 96-associated antigens on major histocompatibility complex class I molecules requires receptor-mediated endocytosis. J Exp Med (2000) 191(11):1965–74. 10.1084/jem.191.11.1965 10839811 PMC2213530

[7] Anderson SL Shen T Lou J Xing L Blachere NE Srivastava PK The endoplasmic reticular heat shock protein gp96 is transcriptionally upregulated in interferon-treated cells. J Exp Med (1994) 180(4):1565–9. 10.1084/jem.180.4.1565 7523574 PMC2191700

[8] Vabulas RM Braedel S Hilf N Singh-Jasuja H Herter S Ahmad-Nejad P The endoplasmic reticulum-resident heat shock protein Gp96 activates dendritic cells via the toll-like receptor 2/4 pathway. J Biol Chem (2002) 277(23):20847–53. 10.1074/jbc.M200425200 11912201

[9] Ito R Shin-Ya M Kishida T Urano A Takada R Sakagami J Interferon-gamma is causatively involved in experimental inflammatory bowel disease in mice. Clin Exp Immunol (2006) 146(2):330–8. 10.1111/j.1365-2249.2006.03214.x 17034586 PMC1942055

[10] Pizarro TT Michie MH Bentz M Woraratanadharm J Smith MF Jr Foley E IL-18, a novel immunoregulatory cytokine, is up-regulated in Crohn’s disease: expression and localization in intestinal mucosal cells. J Immunol (1999) 162(11):6829–35. 10352304

[11] Monteleone G Trapasso F Parrello T Biancone L Stella A Iuliano R Bioactive IL-18 expression is up-regulated in Crohn’s disease. J Immunol (1999) 163(1):143–7. 10384110

[12] Kanai T Watanabe M Okazawa A Nakamaru K Okamoto M Naganuma M Interleukin 18 is a potent proliferative factor for intestinal mucosal lymphocytes in Crohn’s disease. Gastroenterology (2000) 119(6):1514–23. 10.1053/gast.2000.20260 11113073

[13] Monteleone G Biancone L Marasco R Morrone G Marasco O Luzza F Interleukin 12 is expressed and actively released by Crohn’s disease intestinal lamina propria mononuclear cells. Gastroenterology (1997) 112(4):1169–78. 10.1016/S0016-5085(97)70128-8 9098000

[14] Parronchi P Romagnani P Annunziato F Sampognaro S Becchio A Giannarini L Type 1 T-helper cell predominance and interleukin-12 expression in the gut of patients with Crohn’s disease. Am J Pathol (1997) 150(3):823–32. 9060820 PMC1857889

[15] Chikano S Sawada K Shimoyama T Kashiwamura SI Sugihara A Sekikawa K IL-18 and IL-12 induce intestinal inflammation and fatty liver in mice in an IFN-gamma dependent manner. Gut (2000) 47(6):779–86. 10.1136/gut.47.6.779 11076875 PMC1728143

[16] Ishikura T Kanai T Uraushihara K Iiyama R Makita S Totsuka T Interleukin-18 overproduction exacerbates the development of colitis with markedly infiltrated macrophages in interleukin-18 transgenic mice. J Gastroenterol Hepatol (2003) 18(8):960–9. 10.1046/j.1440-1746.2003.03097.x 12859727

[17] Neurath MF Fuss I Kelsall BL Stuber E Strober W. Antibodies to interleukin 12 abrogate established experimental colitis in mice. J Exp Med (1995) 182(5):1281–90. 10.1084/jem.182.5.1281 7595199 PMC2192205

[18] Okamura H Tsutsi H Komatsu T Yutsudo M Hakura A Tanimoto T Cloning of a new cytokine that induces IFN-gamma production by T cells. Nature (1995) 378(6552):88–91. 10.1038/378088a0 7477296

[19] Okamura H Kashiwamura S Tsutsui H Yoshimoto T Nakanishi K. Regulation of interferon-gamma production by IL-12 and IL-18. Curr Opin Immunol (1998) 10(3):259–64. 10.1016/S0952-7915(98)80163-5 9638361

[20] Carson WE Dierksheide JE Jabbour S Anghelina M Bouchard P Ku G Coadministration of interleukin-18 and interleukin-12 induces a fatal inflammatory response in mice: critical role of natural killer cell interferon-gamma production and STAT-mediated signal transduction. Blood (2000) 96(4):1465–73. 10942393

[21] Wirtz S Popp V Kindermann M Gerlach K Weigmann B Fichtner-Feigl S Chemically induced mouse models of acute and chronic intestinal inflammation. Nat Protoc (2017) 12(7):1295–309. 10.1038/nprot.2017.044 28569761

[22] Kliger Y Levy O Oren A Ashkenazy H Tiran Z Novik A Peptides modulating conformational changes in secreted chaperones: from in silico design to preclinical proof of concept. Proc Natl Acad Sci U S A (2009) 106(33):13797–801. 10.1073/pnas.0906514106 19666568 PMC2728974

[23] Wu S Dole K Hong F Noman AS Issacs J Liu B Chaperone gp96-independent inhibition of endotoxin response by chaperone-based peptide inhibitors. J Biol Chem (2012) 287(24):19896–903. 10.1074/jbc.M112.343848 22532561 PMC3370174

[24] Ochayon DE Mizrahi M Shahaf G Baranovski BM Lewis EC Human alpha1-antitrypsin binds to heat-shock protein gp96 and protects from endogenous gp96-mediated injury in vivo. Front Immunol (2013) 4:320 10.3389/fimmu.2013.00320 24191154 PMC3808895

[25] McHenga SS Wang D Li C Shan F Lu C. Inhibitory effect of recombinant IL-25 on the development of dextran sulfate sodium-induced experimental colitis in mice. Cell Mol Immunol (2008) 5(6):425–31. 10.1038/cmi.2008.53 19118508 PMC4072414

[26] Nold MF Nold-Petry CA Zepp JA Palmer BE Bufler P Dinarello CA IL-37 is a fundamental inhibitor of innate immunity. Nat Immunol (2010) 11(11):1014–22. 10.1038/ni.1944 20935647 PMC3537119

[27] Becker C Fantini MC Wirtz S Nikolaev A Kiesslich R Lehr HA In vivo imaging of colitis and colon cancer development in mice using high resolution chromoendoscopy. Gut (2005) 54(7):950–4. 10.1136/gut.2004.061283 15951540 PMC1774595

[28] Kanai T Watanabe M Okazawa A Sato T Yamazaki M Okamoto S Macrophage-derived IL-18-mediated intestinal inflammation in the murine model of Crohn’s disease. Gastroenterology (2001) 121(4):875–88. 10.1053/gast.2001.28021 11606501

[29] Siegmund B Fantuzzi G Rieder F Gamboni-Robertson F Lehr HA Hartmann G Neutralization of interleukin-18 reduces severity in murine colitis and intestinal IFN-gamma and TNF-alpha production. Am J Physiol Regul Integr Comp Physiol (2001) 281(4):R1264–73. 11557635 10.1152/ajpregu.2001.281.4.R1264

[30] Wirtz S Becker C Blumberg R Galle PR Neurath MF. Treatment of T cell-dependent experimental colitis in SCID mice by local administration of an adenovirus expressing IL-18 antisense mRNA. J Immunol (2002) 168(1):411–20. 10.4049/jimmunol.168.1.411 11751987

[31] Rolhion N Barnich N Bringer MA Glasser AL Ranc J Hebuterne X Abnormally expressed ER stress response chaperone Gp96 in CD favours adherent-invasive *Escherichia coli* invasion. Gut (2010) 59(10):1355–62. 10.1136/gut.2010.207456 20587550 PMC2976078

[32] Ng SC Benjamin JL McCarthy NE Hedin CR Koutsoumpas A Plamondon S Relationship between human intestinal dendritic cells, gut microbiota, and disease activity in Crohn’s disease. Inflamm Bowel Dis (2011) 17(10):2027–37. 10.1002/ibd.21590 21910165

[33] Fantuzzi G Puren AJ Harding MW Livingston DJ Dinarello CA. Interleukin-18 regulation of interferon gamma production and cell proliferation as shown in interleukin-1beta-converting enzyme (caspase-1)-deficient mice. Blood (1998) 91(6):2118–25. 9490698

[34] Pena Rossi C Hanauer SB Tomasevic R Hunter JO Shafran I Graffner H. Interferon beta-1a for the maintenance of remission in patients with Crohn’s disease: results of a phase II dose-finding study. BMC Gastroenterol (2009) 9:22. 10.1186/1471-230X-9-22 19302707 PMC2674451

[35] Chen YG Ashok BT Liu X Garikapaty VP Mittelman A Tiwari RK. Induction of heat shock protein gp96 by immune cytokines. Cell Stress Chaperones (2003) 8(3):242–8. 10.1379/1466-1268(2003)008<0242:IOHSPG>2.0.CO;2 14984057 PMC514877

[36] Neurath MF. Cytokines in inflammatory bowel disease. Nat Rev Immunol (2014) 14(5):329–42. 10.1038/nri3661 24751956

[37] Strober W Fuss IJ. Proinflammatory cytokines in the pathogenesis of inflammatory bowel diseases. Gastroenterology (2011) 140(6):1756–67. 10.1053/j.gastro.2011.02.016 21530742 PMC3773507

[38] Caruso R Sarra M Stolfi C Rizzo A Fina D Fantini MC Interleukin-25 inhibits interleukin-12 production and Th1 cell-driven inflammation in the gut. Gastroenterology (2009) 136(7):2270–9. 10.1053/j.gastro.2009.02.049 19505427

[39] Colombel JF Rutgeerts P Malchow H Jacyna M Nielsen OH Rask-Madsen J Interleukin 10 (Tenovil) in the prevention of postoperative recurrence of Crohn’s disease. Gut (2001) 49(1):42–6. 10.1136/gut.49.1.42 11413109 PMC1728363

[40] Schreiber S Fedorak RN Nielsen OH Wild G Williams CN Nikolaus S Safety and efficacy of recombinant human interleukin 10 in chronic active Crohn’s disease. Crohn’s Disease IL-10 Cooperative Study Group. Gastroenterology (2000) 119(6):1461–72. 10.1053/gast.2000.20196 11113067

[41] Fedorak RN Gangl A Elson CO Rutgeerts P Schreiber S Wild G Recombinant human interleukin 10 in the treatment of patients with mild to moderately active Crohn’s disease. The Interleukin 10 Inflammatory Bowel Disease Cooperative Study Group. Gastroenterology (2000) 119(6):1473–82. 10.1053/gast.2000.20229 11113068

[42] Herrlinger KR Witthoeft T Raedler A Bokemeyer B Krummenerl T Schulzke JD Randomized, double blind controlled trial of subcutaneous recombinant human interleukin-11 versus prednisolone in active Crohn’s disease. Am J Gastroenterol (2006) 101(4):793–7. 10.1111/j.1572-0241.2005.00356.x 16635225

[43] Reinisch W Hommes DW Van Assche G Colombel JF Gendre JP Oldenburg B A dose escalating, placebo controlled, double blind, single dose and multidose, safety and tolerability study of fontolizumab, a humanised anti-interferon gamma antibody, in patients with moderate to severe Crohn’s disease. Gut (2006) 55(8):1138–44. 10.1136/gut.2005.079434 16492717 PMC1856289

[44] Hueber W Sands BE Lewitzky S Vandemeulebroecke M Reinisch W Higgins PD Secukinumab, a human anti-IL-17A monoclonal antibody, for moderate to severe Crohn’s disease: unexpected results of a randomised, double-blind placebo-controlled trial. Gut (2012) 61(12):1693–700. 10.1136/gutjnl-2011-301668 22595313 PMC4902107

[45] Derkx B Taminiau J Radema S Stronkhorst A Wortel C Tytgat G Tumour-necrosis-factor antibody treatment in Crohn’s disease. Lancet (1993) 342(8864):173–4. 10.1016/0140-6736(93)91375-V 8101267

[46] van Dullemen HM van Deventer SJ Hommes DW Bijl HA Jansen J Tytgat GN Treatment of Crohn’s disease with anti-tumor necrosis factor chimeric monoclonal antibody (cA2). Gastroenterology (1995) 109(1):129–35. 10.1016/0016-5085(95)90277-5 7797011

[47] Peyrin-Biroulet L Lemann M. Review article: remission rates achievable by current therapies for inflammatory bowel disease. Aliment Pharmacol Ther (2011) 33(8):870–9. 10.1111/j.1365-2036.2011.04599.x 21323689

