# Supplementary figures and images for: Gp96 Peptide Antagonist gp96-II Confers Therapeutic Effects in Murine Intestinal Inflammation
Source: Front Immunol. 2017 Dec 11;8:1531. doi: 10.3389/fimmu.2017.01531 (PMC5732239; doi:10.3389/fimmu.2017.01531)

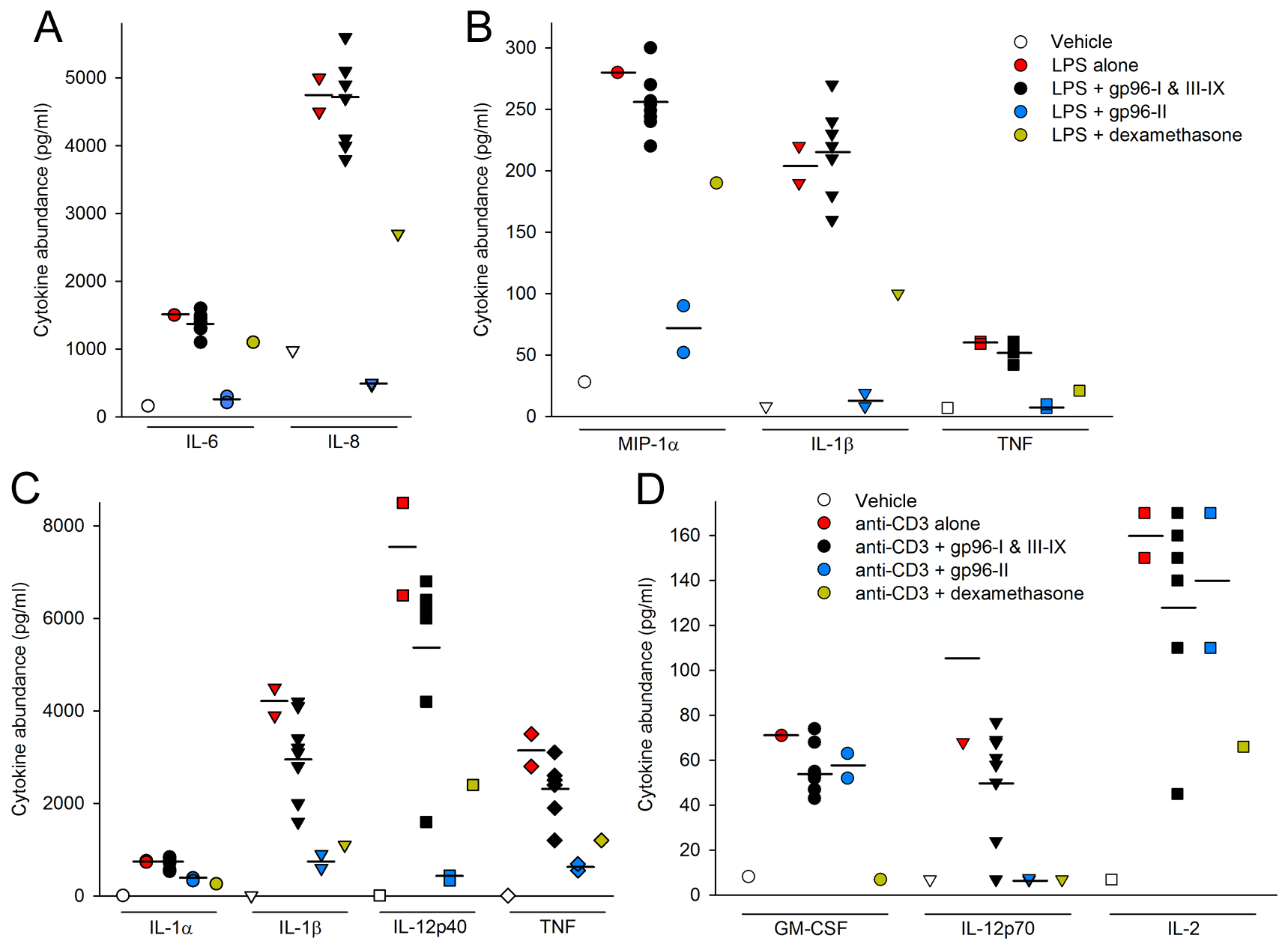

Supplement: Figure S1 — Anti-inflammatory activity of nine gp96-II peptides. gp96-II peptide (30 µM, open symbols) inhibits the production of cytokines from human peripheral blood mononuclear cells in response to a 24-h treatment with 1 µg/ml lipopolysaccharide (LPS) (A,B) or 48 h with 30 µg/ml anti-CD3 (C,D) in comparison to peptides gp96-I and III-X (30 µM, solid symbols) and dexamethasone. Individual cytokine abundance is depicted with each symbol representing one biological replicate. Horizontal lines show means. [file Image_1.TIF]
