# Supplementary material for: Gp96 Peptide Antagonist gp96-II Confers Therapeutic Effects in Murine Intestinal Inflammation
Source: Front Immunol. 2017 Dec 11;8:1531. doi: 10.3389/fimmu.2017.01531 (PMC5732239; doi:10.3389/fimmu.2017.01531)
Supplement: Table S1 — Stool consistency: normal stool, stool with an appearance of well-formed pellets; slightly loose stool, loose stool with pasty, semi-formed, soft materials that do not adhere to anal fur; diarrhea, liquid stool that adheres to anal fur. Gross bleeding, an appearance of visible blood adhering to anal fur. [file Table_1.DOCX]

|  | **Vehicle** | | | | Gp96-II peptide | | | |
| --- | --- | --- | --- | --- | --- | --- | --- | --- |
| **Animal #** | **Weight-loss %** | **Weight-loss score** | **Stool**  **consistency** | **Fecal blood** | Weight-loss % | Weight-loss | Stool consistency | Fecal  blood |
| 1 | 15 | 3 | 3 | 2 | 6 | 2 | 2 | 1 |
| 2 | 10 | 3 | 3 | 2 | 4 | 1 | 0 | 0 |
| 3 | 11 | 3 | 3 | 2 | 5 | 1 | 2 | 1 |
| 4 | 9 | 2 | 3 | 1 | 6 | 2 | 2 | 1 |
| 5 | 8 | 2 | 2 | 2 | 5 | 1 | 0 | 1 |
| 6 | 7 | 2 | 2 | 1 | 4 | 1 | 0 | 0 |
| 7 | 8 | 2 | 3 | 2 | 5 | 1 | 2 | 0 |
| 8 | 7 | 2 | 3 | 1 | 7 | 2 | 2 | 2 |
| 9 | 7 | 2 | 3 | 1 | 4 | 1 | 0 | 0 |
| 10 | -- | -- | -- | -- | 5 | 1 | 0 | 1 |
| **Average** | **9.11** | **2.33** | **2.78** | **1.56** | **5.10** | **1.30** | **1.00** | **0.70** |
